# Ionic-exchange immobilization of ultra-low loading palladium on a rGO electro-catalyst for high activity formic acid oxidation

**DOI:** 10.1039/c8ra03043j

**Published:** 2018-05-22

**Authors:** Jiuxiao Sun, Xingying Luo, Weiwei Cai, Jing Li, Zhao Liu, Jie Xiong, Zehui Yang

**Affiliations:** College of Materials Science and Engineering, Wuhan Textile University Fangzhi RD Wuhan 430200 China; Sustainable Energy Laboratory, Faculty of Materials Science and Chemistry, China University of Geosciences (Wuhan) 388 Lumo Road 430074 Wuhan P. R. China willcai1985@gmail.com caiww@cug.edu.cn yeungzehui@gmail.com

## Abstract

A formic acid oxidation electro-catalyst with ultra-low palladium (Pd) loading was prepared *via* an ionic exchange method by utilizing the acidic functional groups on graphene oxide (GO). After simultaneous reduction of exchanged Pd^2+^ and residual functional groups on the GO surface, an ionic exchange reduced Pd catalyst supported on reduced GO (IE-Pd/rGO) was obtained. Three times improved formic acid oxidation mass activity compared with that of the conventional synthesized Pd/C catalyst was exhibited for the IE-Pd/rGO catalyst. More importantly, formic acid oxidation stability on the IE-Pd/rGO catalyst was remarkably improved due to synergistic effect of the strong immobilization of Pd nanoparticles and the effect of *in situ* doped N on the rGO support.

## Abstract 2

A formic acid oxidation electro-catalyst with ultra-low palladium (Pd) loading was prepared *via* an ionic exchange method by utilizing the acidic functional groups on graphene oxide (GO).

## Graphical abstract

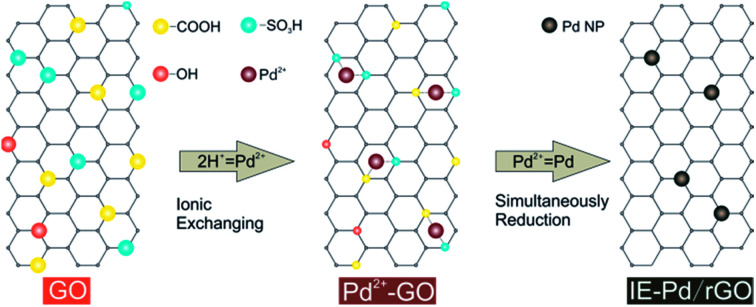

## 1. Introduction

Formic acid (FA) has been considered as one of the most potential alternative liquid fuels to replace methanol in direct liquid fuel cells due to the much faster kinetics of the FA oxidation reaction (FAOR) than that of the methanol oxidation reaction^1–4^ and much lower fuel crossover rate through the proton exchange membrane.^5,6^ Despite the development on Pt based FAOR catalysts,^7–10^ noble metal palladium (Pd) electro-chemical catalysts^11–13^ are required for FA oxidation catalysis to achieve satisfactory power output of the direct formic acid fuel cell (DFAFC).^14–22^ Other than the high cost of Pd, poor FAOR stability on Pd catalyst is also an important issue strongly hinders the practical application of DFAFC technology.^23,24^ In order to overcome this stability issue, numerous strategies were carried out to alter the FAOR process or to improve the efficiency of single Pd active sites.^25,26^ Among these strategies, doping a second metal with Pd to change the FAOR process is the most commonly used one.^27–30^ Due to the electron effect^31^ or the third-body effect,^32,33^ generation of Pd poison intermediates can be suppressed. Most mechanism studies claimed that continuous Pd active sites should be isolated no matter what the poison intermediate was.^34–36^ An alternative strategy is to stabilize the Pd nanoparticles isolated during the DFAFC operation.^36^ The best route to stably isolate the Pd nanoparticles is mono-dispersing the nanoparticles on graphene,^37–39^ which has been widely studied as catalyst support^40^ due to the great electronic conductivity and abundant surface functional groups on graphene oxide (GO).^41–43^ Plenty of Pd/graphene catalysts were therefore developed with improved FAOR catalytic performance than the conventional Pd/C catalysts.^44,45^ However, Pd loading in the reported Pd/graphene catalysts is still high.^39^

**Fig. 1:**
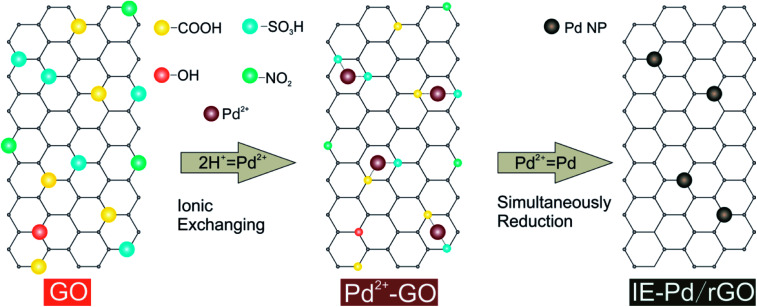
Synthesis route of the IE-Pd/rGO catalyst.

In the present work, a novel route (Fig. 1) was proposed to synthesis highly active Pd loaded reduced GO (rGO) catalyst with ultra-low palladium loading. Acidic functional groups, including –COOH and –SO_3_H, generated on GO surface was used for Pd^2+^ exchange during this ionic-exchange assisted synthesis process. The exchanged Pd^2+^ on GO surface was subsequently *in situ* reduced together with the residual oxidation functional groups, *e.g.* –OH and NO_2_ groups. Pd nanoparticles were therefore stably anchored on rGO to produce an ionic exchange assisted Pd/rGO (IE-Pd/rGO) catalyst. The IE-Pd/rGO catalyst exhibited great catalytic efficiency and stability for FAOR compared with the conventional prepared Pd/C catalyst. As a result, with only *ca.* 7 wt% Pd loaded, the IE-Pd/rGO FAOR catalyst performed much better than the conventional Pd/C with more than 20 wt% Pd loaded in terms of both electrochemical oxidation current and stability.

## 2. Experimental

### 2.1. Catalyst synthesis

#### 2.1.1. IE-Pd/rGO catalyst

GO was prepared from oxidation of powdered flake graphite using the modified Hummers' method.^46^ The exchange of Pd^2+^ ions of the functionalized GO was achieved by dispersing GO (0.1 g) in deionized water (50 mL). Subsequently, palladium acetate (45 mmol) was added into the dispersion and the mixture was treated in an ultrasonic bath for 30 min. The mixture was then magnetically stirred overnight for Pd^2+^ exchange. Despite the low solubility of palladium acetate in water, ionic exchange would take place completely due to the continuously consumption of palladium acetate. The Pd^2+^ exchanged GO sheets were dried at 40 °C for 12 h in vacuum oven subsequent to washing with deionized water and acetone for several times. Simultaneously reduction of Pd^2+^ and oxygen-contained groups on the Pd^2+^/GO sheets were carried out by adding excessive hydrazine hydrate (85% N_2_H_5_OH aqueous solution) into 40 mL Pd^2+^-GO solution. After being stirred overnight, the black slurry was filtered, washed and dried at 60 °C. The product was denoted as IE-Pd/rGO for further use.

#### 2.1.2. IR-Pd/C catalyst

The Pd/C catalyst for comparison was prepared by conventional impregnation reduction method and denoted as IR-Pd/C catalyst.^47^ 100 mg Vulcan XC-72 carbon black and 10 mL H_2_PdCl_4_ (2.5 mg mL^−1^) solution were dispersed uniformly in 100 mL of deionized water with vigorously stirring for 1 h. 15 mL freshly prepared 20 mM cetyltrimethyl ammonium bromide (CTAB) solution, 5 mL 50 mM ascorbic acid solution and 5 mL 0.5 M NaOH were added into the mixture in sequence with magnetic stirring. After reaction overnight, the solution was filtered, and thoroughly washed by hot deionized water to get rid of CTAB. Finally, the catalyst was dried at 60 °C and was ground into a fine powder state.

### 2.2. Characterization

Powder X-ray diffraction (XRD) analyses were performed on a Bruker D8-FOCUS Advance diffractometer with Cu Kα radiation (*λ* = 1.540598 Å). The average crystal size of the Pd nanoparticles was subsequently calculated using the Scherrer equation (*D* = 0.9*λ*/*β* cos *θ*, where *λ* = 1.540598 Å is the wavelength of the X-ray, *β* is the full-width at half-maximum height and *θ* is the diffraction angle).^48^ Surface morphology of the prepared catalyst was characterized by scanning electron microscopy (SEM) on a Hitachi-SU8010 microscope. Transmission electron microscopy (TEM) analysis on the catalysts was carried out on Tecnai G220 microscope. The surface composition of the IE-Pd/rGO catalyst was analyzed using a ULVAC-PHI Quantera II photoelectron spectroscopy (XPS) system with Al Kα radiation (*hν* = 1486.6 eV). The thermogravimetric (TG) analysis was carried out on a STA449F3 thermogravimetric analyzer under an O_2_ atmosphere from 30 to 800 °C at a heating rate of 10 °C min^−1^. Pd loading of the catalysts was calculated from the residual weight ratio at 800 °C of the sample (*x*_Pd_ = *w*_re_*M*_Pd_/*M*_PdO_, where *w*_re_ is the residual weight ratio of the sample, *M*_Pd_ and *M*_PdO_ are the molecular weight of Pd and PdO, respectively).

### 2.3. Electrochemical measurements

Electro-catalytic activities of the prepared catalysts were measured in a three-electrode cell using a VMP3 electrochemical workstation. The three-electrode electrochemical cell using a glassy carbon (GC) electrode (3 mm in diameter) coated with catalyst serving as the working electrode, a saturated calomel electrode (SCE) and a graphite rod as the reference electrode and the counter electrode, respectively. The catalyst inks were prepared by ultrasonically mixing of catalyst (4 mg), ethanol (0.20 mL, 99 wt%), Nafion solutions (0.05 mL, 5 wt%) and deionized water (0.75 mL). After evenly dispersed, 10 μL of the suspension was drop dipped onto the GC electrode and subsequently used as the working electrode in measurements. This was repeated once so that it had a loading of 0.56617 mg cm^−2^ catalyst, and the coating was dried at room temperature for 30 min.

Cyclic voltammetry (CV) measurements were carried out in N_2_-saturated 0.5 M H_2_SO_4_ at a scanning rate of 20 mV s^−1^. Electrochemical surface area (ECSA) of the catalyst can be calculated from charges (*Q*) involved in the hydrogen adsorption processes according to the equation of ECSA = *Q*/0.42 mC cm^−2^ × *M*_Pd_,^3,49^ where *M*_Pd_ is the Pd loading on the working electrode, 0.42 mC cm^−2^ is the assuming electrical charge associated with the monolayer adsorption of hydrogen on Pd. Linear sweep voltammetry (LSV) and chronoamperometry (CA) measurements were carried out in a 0.5 M H_2_SO_4_ + 0.5 M HCOOH solution. All electrochemical experiments were performed at room temperature.

## 3. Results and discussion

**Fig. 2:**
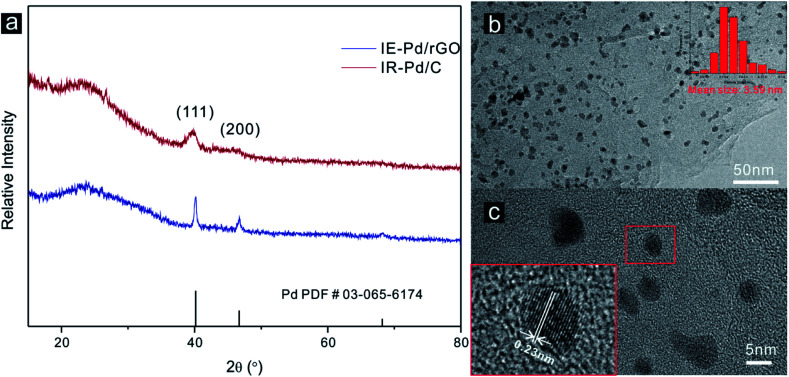
(a) XRD spectra of the IE-Pd/rGO catalyst and the IR-Pd/C catalyst; (b) TEM image of the IE-Pd/rGO catalyst (inset: size distribution of Pd nanoparticles in the IE-Pd/rGO catalyst) and (c) HRTEM image of the IE-Pd/rGO catalyst.

Characteristic peaks of (111) and (200) of Pd metal can be detected from the XRD spectrum of the IE-Pd/rGO catalyst as displayed in Fig. 2a, agree well with the PDF card: 03-065-6174. Much boarder (111) peak in the XRD spectrum of the IE-Pd/rGO catalyst than that of the IR-Pd/C catalyst, which was synthesized from a traditional impregnation reduction method, indicates the smaller size of the IE-Pd/rGO catalyst. The board peak around the 2*θ* = 22° is attributed to the X-ray diffraction on the rGO sheets. TEM image on the IE-Pd/rGO catalyst (Fig. 2b) confirms that the Pd nanoparticles are uniformly dispersed on rGO surface with average size of *ca.* 3.59 nm, agreeing well with that calculated from the (111) peak of the XRD spectrum. It can be also revealed from the TEM images that the Pd nanoparticles are uniformly anchored on the rGO surface with large distance between each two nanoparticles, which is attributed to the diluted distribution of acid groups on the GO sheets. At the same time, the crystal domains within the Pd nanoparticles have an interfringe distance of 0.23 nm (Fig. 2c), close to the lattice spacing of the (111) planes of the face-centered cubic (fcc) Pd crystal, indicating a stable catalytically activity of the IE-Pd/rGO catalyst.^50^

**Fig. 3:**
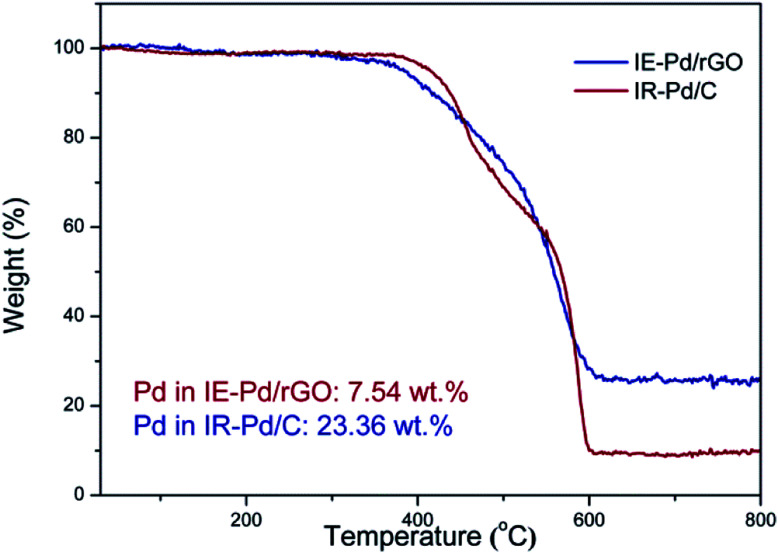
TG curves of the IE-Pd/rGO catalyst and the IR-Pd/C catalyst.

To evaluate the Pd loading in the IE-Pd/rGO catalyst, TG analysis was carried out on the IE-Pd/rGO catalyst in O_2_ condition. According to the TG curves as shown in Fig. 3, Pd loading of the IR-Pd/C catalyst was calculated to be 23.36 wt%, agreeing well with the Pd : C ratio in the precursors. In contrast, Pd loading of the IE-Pd/rGO catalyst is low to 7.54 wt%, only 1/3 of that of the Pd/C catalyst. This value is slightly higher than desired (*ca.* 5 wt%) due to the loss of small pieces of GO/rGO sheets during the catalyst fabrication.

**Fig. 4:**
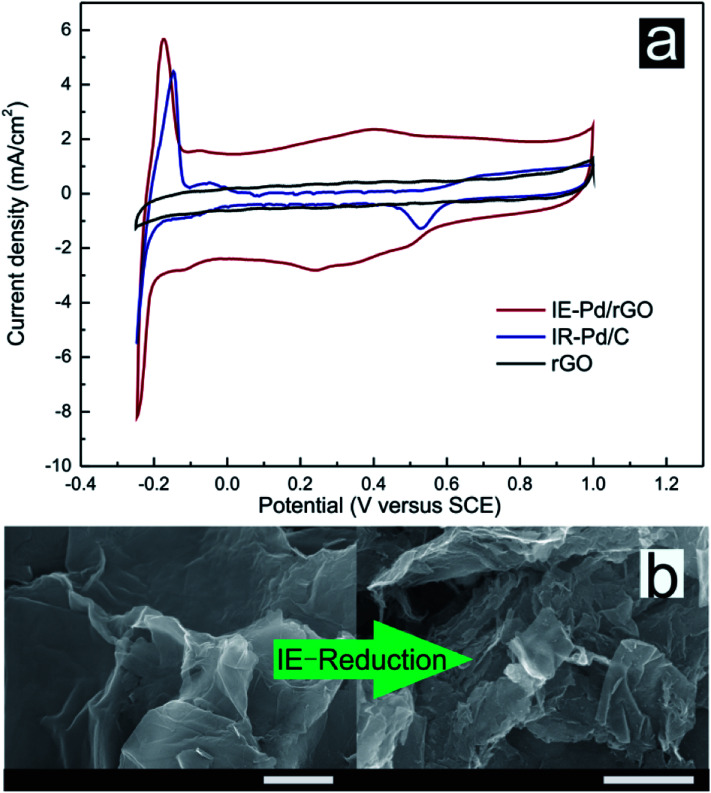
(a) CV curves of the IE-Pd/rGO catalyst, the IR-Pd/C catalyst and rGO in 0.5 M H_2_SO_4_ solution. (b) SEM images of original rGO and the IE-Pd/rGO catalyst (scale bar: 1 μm).

0.5 M H_2_SO_4_ solution was subsequently used as electrolyte for CV measurements of both the IE-Pd/rGO catalyst and the IR-Pd/C catalyst to evaluate the electrochemical activity area of the catalysts. Although Pd loading of the IE-Pd/rGO catalyst is much lower compared with the IR-Pd/C catalyst, ECSA of the IE-Pd/rGO catalyst (89.8 m^2^ g^−1^) is almost three times higher than that of the IR-Pd/C catalyst according to the CV curves displayed in Fig. 4a. Moreover, capacitance of the IE-Pd/rGO catalyst was significantly enlarged compared to that of the original rGO, on where no proton adsorption/desorption peak can be detected, due to that the multilayered rGO nanosheets were further stripped and were broken to small pieces. As demonstrated in Fig. 4b, size of the rGO nanosheets were significantly reduced after the IE-reduction synthesis. The size reduction on rGO layer would result in higher active surface area for electrochemical reaction. The enlarged surface area also ensures that the nanoparticles are far away from each other and won't aggregate during the fuel cell operation.

**Fig. 5:**
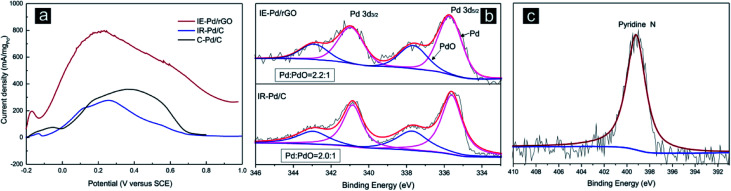
(a) Linear sweep voltammetry (LSV) curves in 0.5 M H_2_SO_4_ + 0.5 M HCOOH solution on the IE-Pd/rGO catalyst, the IR-Pd/C catalyst and the C–Pd/C catalyst; (b) XPS spectra of Pd 3d_3/2_ and Pd_5/2_ of the IE-Pd/rGO catalyst and the IR-Pd/C catalyst; (c) XPS spectrum of N 1s in the IE-Pd/rGO catalyst.

Electrochemical FAOR catalytic activity of the IE-Pd/rGO catalyst was further investigated with the linear sweep voltammetry (LSV) curve compared with that of the IR-Pd/C catalyst and the commercial 20% Pd/C (C–Pd/C, Alfa Aesar) catalyst in Fig. 5a. The IE-Pd/rGO catalyst exhibited much high current density during the entire potential sweep from −0.2 V to 1.0 V. Peak FAOR current density on the IE-Pd/rGO catalyst is high than 800 mA mg_Pd_^−1^, which is *ca.* 4 times higher than that of the IR-Pd/C catalyst as well as that of the C–Pd/C catalyst. This mass activity is among the best reported Pd based FAOR catalysts at the identical condition.^20,37,51,52^ Despite the ultra-low Pd loading in the IE-Pd/rGO catalyst, Pd(0) : Pd(ii) ratio in the IE-Pd/rGO catalyst is higher than that of the IR-Pd/C catalyst as demonstrated in the XPS spectra in Fig. 5b. Other than the enhanced FAOR activity, FAOR potential of the IE-Pd/rGO catalyst is also negatively shifted by 40 mV compared with the IR-Pd/C catalyst due to the assistant of doped N atoms on rGO surface, which can be confirmed by XPS spectrum of N 1s on the IE-Pd/rGO catalyst in Fig. 5c, resulted from the N-containing functional groups, *e.g.* –NO_2_, on GO surface.^53–57^ Specifically, specific activity of the IE-Pd/rGO catalyst is calculated to be 0.9 mA cm_Pd_^−2^ by considering the mass activity and ECSA, which similar to that of the reported Pd/C catalysts^18^ and the IR-Pd/C catalyst, indicating that FAOR process on IE-Pd/rGO catalyst was not altered.

**Fig. 6:**
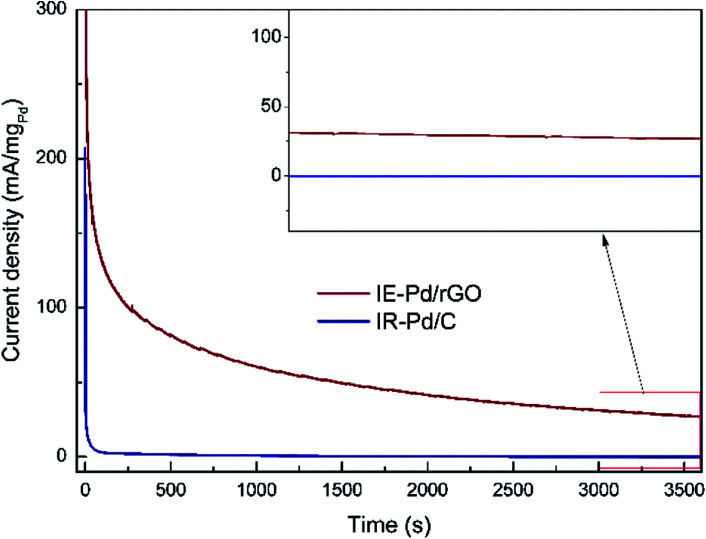
Chronoamperometry (CA) curves of the IE-Pd/rGO catalyst and the IR-Pd/C catalyst. (inset: magnified CA curves from 3000 to 3600 s).

Due to the synergistic effect of Pd uniformly dispersion and N atomic doping, stability of FAOR on the IE-Pd/rGO catalyst was significantly improved. The IR-Pd/C catalyst exhibited an extremely poor stability as shown in the CA curve in Fig. 6. FAOR current density was quickly deceased to *ca.* 0 within 100 seconds. As a comparison, FAOR current density of the IE-Pd/rGO catalyst was slowly decreased during the entire 3600 s CA measurement. According to the magnified curves shown in the inset of Fig. 6, FA oxidation current density on the IE-Pd/rGO catalyst can be stabilized at *ca.* 30 mA mg_Pd_^−1^ after the 3600 s CA measurement.

## 4. Conclusions

In summary, an ionic exchange strategy was developed for immobilization of Pd nano-particles on rGO support to achieve ultra-low Pd loading for efficient catalysis of formic acid electro-oxidation. Pd^2+^ ions were exchanged with the protons in the acid functional groups on GO surface and highly dispersed Pd nano-particles were firmly loaded rGO after mild reduction. Three times higher FA electro-oxidation mass activity was therefore obtained compared with the conventional impregnation method synthesized Pd/C catalyst. More importantly, FA oxidation stability on this IE-Pd/rGO catalyst was also strongly improved due to the strong immobilization of Pd nano-particle and the effect of *in situ* doped N from the N-containing functional groups on GO.

## Author contributions

Jiuxiao Sun and Xingying Luo contribute equally to this work. The manuscript was written through contributions of all authors. All authors have given approval to the final version of the manuscript.

## Conflicts of interest

There are no conflicts to declare.

## Supplementary Material
